# Association between needle track bleeding and postoperative immediate pneumothorax in CT-guided percutaneous transthoracic lung biopsies: a cross-sectional study

**DOI:** 10.1038/s41598-023-44560-2

**Published:** 2023-11-01

**Authors:** Shao-Quan Zhou, Fang Luo, Kang Li, Xiong Ran, Fu-Rong Lv

**Affiliations:** 1 The First Affiliated Hospital of Chongqing Medical University, No. 1 Youyi Road, Yuanjiagang, Yuzhong District, Chongqing, 400016 China; 2 The Chongqing Traditional Chinese Medicine Hospital, Chongqing Academy of Traditional Chinese Medicine, Chongqing, China; 3 Chongqing General Hospital, Chongqing, China

**Keywords:** Risk factors, Medical imaging

## Abstract

The relationship between Needle Track Bleeding (NTB) and the occurrence of postoperative immediate pneumothorax remains unclear. In our cross-sectional study, we conducted a retrospective collected of data from 674 consecutive patients who underwent CT-guided percutaneous transthoracic lung biopsies between 2019 and 2022. A logistic regression model was employed to explore the association between NTB and postoperative immediate pneumothorax, and restricted cubic spline curves was used to investigate the link and its explicit curve shape. A sensitivity analysis was performed by transforming the continuous NTB into categorical variable and calculated an E-value. A total of 453 participants (47.90% male) were included in our analysis. The postoperative immediate pneumothorax rate was 41.05% (186/453). We found a negative correlation between NTB and postoperative immediate pneumothorax (OR = 0.91, 95%CI 0.88–0.95) after adjusting for confounding factors. This relationship was nonlinear, with a key inflection point at NTB of 8 mm. No significant link was noted for NTB > 8 mm (OR = 0.98, 95%CI 0.95–1.02), while a protective association was observed for NTB ≤ 8 mm (OR = 0.74, 95%CI 0.66–0.81). NTB showed a nonlinear, protective correlation with postoperative immediate pneumothorax. However, when NTB exceeded 8 mm, the protective association was not observed.

## Introduction

Computed tomography-guided percutaneous transthoracic lung biopsy (CT-PTLB) is a well-established, minimally invasive procedure that offers significant diagnostic value in procuring pulmonary tissue samples^1^. Despite its recognized safety and effectiveness, pneumothorax and pulmonary hemorrhage, the most common complications, still pose significant challenges for clinicians.

Pneumothorax, characterized by air leak into the chest cavity, occurs in approximately 9–54% of patients undergoing CT-PTLB, with an average incidence rate about 20%^2^. Additionally, 2–15% of these incidents necessitate the insertion of chest tube^3,4^. Severe pneumothorax cases can pose life-threatening risks, induce patient discomfort^5^, prolong hospital stays^6^, and inflate healthcare costs^7^.

Pulmonary hemorrhage, defined as the emergence of new opacity surrounding the biopsied lesion^8^, with reported frequencies ranging from 4 to 27%^8,9^. It can be classified into needle track bleeding (NTB), and around lesion bleeding^10^.This classification offers a vital tool for estimating the hemorrhage severity and predicting patient prognosis.

Interestingly, accumulating research suggests a potential link between NTB and the incidence of postoperative pneumothorax. For instance, Massimo De Filippo et al.^10^ indicated that the presence of NTB over 6 mm correlates with a lower occurrence of pneumothorax. Similar findings were reported by Vittorio Sabatino et al.^11^, asserting that NTB exceeding 6 mm could serve as a protective factor against pneumothorax development (OR = 0.503) and subsequent requirement for chest tube insertion (OR = 0.416). However, the cut-off value appears to be a controversy and perhaps arbitrarily determined, its rationality is not fully explained.

Moreover, a recent study by Esra Soylu et al.^12^ proposed that NTB might act as a protective effect against pneumothorax, Nevertheless, the study lacked a comprehensive elucidation of the underlying mechanism. Given these existing uncertainties and gap in the field, further investigations the interaction between NTB and pneumothorax are needed.

Remarkably, no study to date has investigated the possibility of a non-linear relationship between NTB and postoperative immediate pneumothorax. Thus, we hypothesis is that a nonlinear link between NTB and postoperative immediate pneumothorax. Elucidating this association would not only enhance our understanding of the impact of NTB on pneumothorax but could also facilitate strategies for preventing or reducing immediate postoperative pneumothorax. Furthermore, our findings may influence clinical practice by offering robust evidence for efficacious biopsy tract closure strategies.

## Material and methods

### study population

This cross -sectional study included adult, non-pregnant patients who consecutive underwent CT-TPLB at our hospital between January 1, 2019, and November 22, 2022. The Chongqing General Hospital Ethic Committee approved this retrospective study (Ethics 28 Committee of Chongqing General Hospital, ID: XJS S2022-052-01). Given the anonymous nature of the data, the Ethic Committee of Chongqing General Hospital determined that individual patient consent was not required. All human procedures were performed in accordance with the Declaration of Helsinki of 1964 and its subsequent amendments. The study was conducted according to the STROCSS 2021 guidelines^13^. Patients’ demographic and clinical data were acquired from their medical records, while biopsy-related data were sourced from the Picture Archiving and Communication System (PACS, Kodak Carestream, Rochester, NY) and Philips’ IntelliSpace Portal workstation.

**Figure 1:**
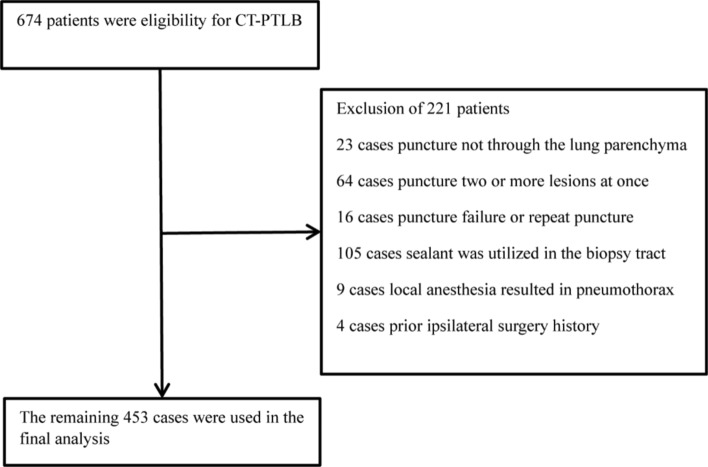
Flowchart of the participants selection.

Among 674 patients, individuals were excluded if (1) puncture not through the pulmonary parenchyma (n = 23), (2) puncture two or more lesions at once (n = 64), (3) puncture failure or repeat puncture (n = 16), (4) the biopsy track was closed using sealant (n = 105), (5) local anesthesia resulted in pneumothorax (n = 9), and (6) prior ipsilateral surgery history (n = 4). Ultimately, a total of 453 patients incorporated in this study (Fig. 1).

### Outcome

The primary outcome of this study is to investigate the occurrence of post-operative immediate pneumothorax. It is defined as the development of pneumothorax after withdrawal of the puncture needle but before the patient is discharged from the operating room, confirmed by CT scan. We also examine the intraoperative pneumothorax, which manifests before the withdrawal of the puncture needle, and delayed pneumothorax characterized by the emergence of pneumothorax symptoms or the need for intervention within a seven-day post-operative period, after the patient has left the operating room, most cases are confined through chest X-ray examinations.

A seasoned medical expert, who specializes in the diagnosis of thoracic diseases, blind to NTB, review of the puncture CT images vis the PACS software. The reviewer evaluates for the presence or absence of pneumothorax and measured other biopsy-related variables besides NTB, with detailed and accurate records.

### Main exposure

Biopsy puncture images were reviewed by another medical imaging expert specializing in chest disease diagnosis. The images were analyzed using Philips' IntelliSpace Portal workstation, set to Lung window (window width: 1600 Hu, window level: − 600Hu) with a 2 mm slice thickness and 2 mm inter-slice distance.

**Figure 2:**
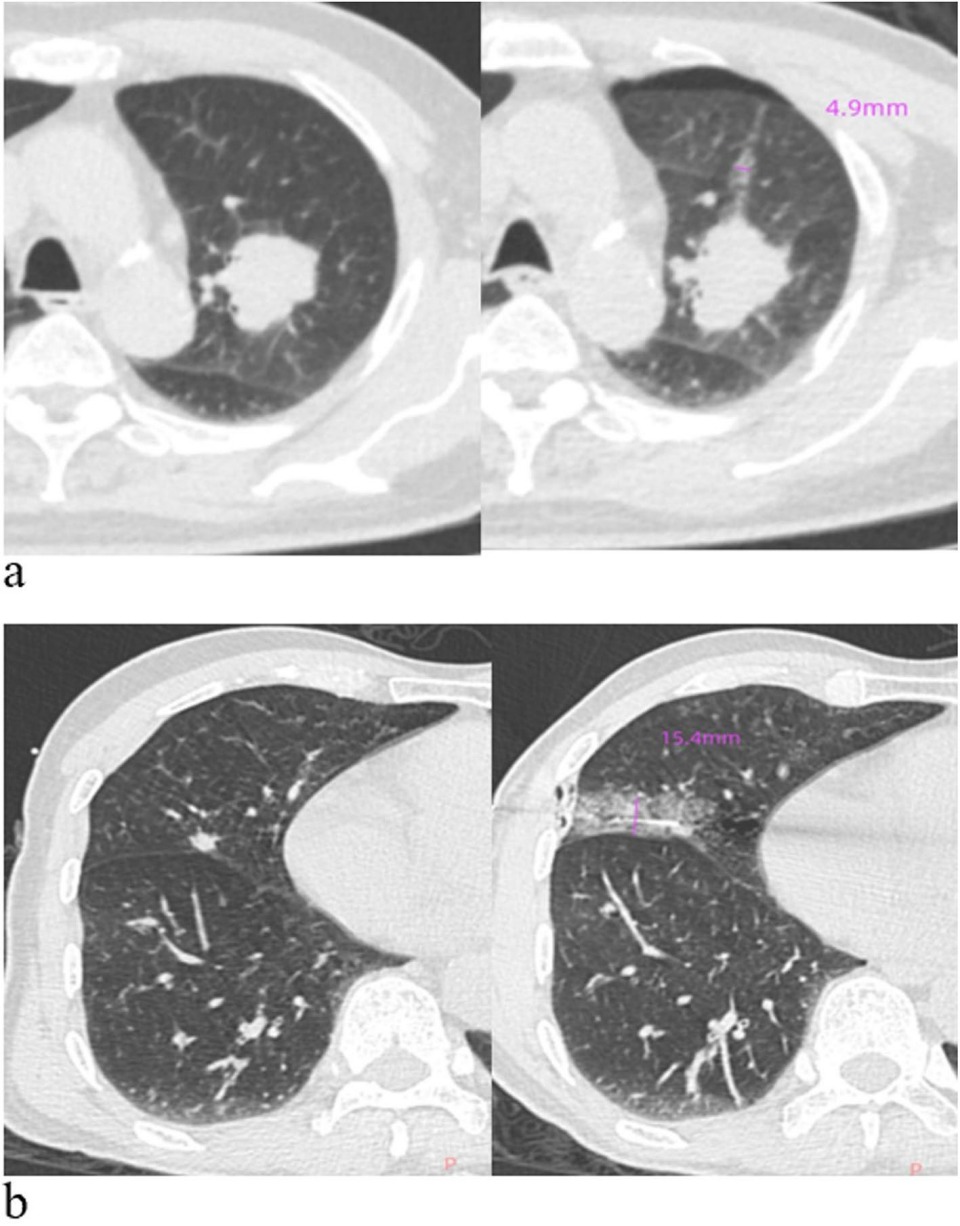
Needle track blooding (NTB) width measurement. (**a**) NTB with minor pneumothorax: A 4.9 mm ground-glass opacity along the needle path is seen post-biopsy after CT-PTLB on a left upper lobe mass, diagnosed as Adenocarcinoma. (**b**) NTB without pneumothorax: post-biopsy shows a 15.4 mm ground-glass and consolidative opacity after CT-TPLB on right middle lobe nodule, with no pneumothorax, diagnosed also as lung adenocarcinoma.

NTB manifests as pulmonary hemorrhage along the needle trajectory, confirmed by post-biopsy CT revealing new consolidative or ground-glass opacities (Fig. 2). A critical aspect of this investigation involved the quantitative evaluation of the width of NTB across all patients, regardless of the presence of pneumothorax. This measurement was carried out employing a straight-line tool on the last axial CT images and documented the maximal width of NTB at three distinct time intervals. An average maximal width was then calculated from these measurements, retaining only integer results for subsequent analysis.

### Covariates

Covariates were certain factors based on hypothesized to be associated with NTB or pneumothorax and thorough review of the existing literature. The collected covariates consisted of socio-demographics, lesion characteristics, and procedural factors.

Socio-demographic variables comprised age, sex (female, male), height, weight, blood pressure (both systolic and diastolic), smoking status (never smoker, former ≥ 1 years, current or former < 1 years), alcohol consumption (never drinking, former ≥ 1 years, current or former < 1 years), and chronic obstructive pulmonary disease (COPD).

Covariates related to the lesion factored in its localization within the lung and its corresponding lobes, as well as lesion's diameter (along the maximum long-axis diameter).

Procedure-related covariates included the transpulmonary needle path length (the distance of aerated lung measured along needle from the pleural puncture site to the edge of the lung lesion), the entire needle path length (the distance along needle from the skin puncture site to the edge of the lung lesion), the needle-pleura angle (defined as the angle between the longitudinal axis of the needle and a line perpendicular to the tangent at the point where the needle contacts the pleura)^14^, the operation duration (defined as the time from insertion puncture needle to the completion of the final CT scan before the patient leaves the operating room), the number of pleural punctures and needle redirections, the operator experience, the interlobar fissure puncturing, the patient’s puncture position, along needle track CT attenuation values (measured using an adjustable rectangular region of interest (ROI) on pre-puncture localization images, with a transverse diameter of about 5–10 mm and length set by lung puncture depth to avert partial volume effects ), the gauge of needle, and the pathological results.

### Biopsy protocol

All patients underwent preoperative blood tests to ensure appropriate platelet counts and clotting times. A review of patient medical history and a preoperative chest CT-enhanced scan were conducted.

Patient positioning—supine, prone, or lateral decubitus—was determined to the lesion location and patient comfort. All patients were awake during the procedures. An optimal puncture route was chosen by radiologists to minimize biopsy tract length and avert complications. Sterile technique was applied, and 2% lidocaine used for local anesthesia. A 17-gauge guiding needle was inserted into the lesion, followed by an 18-gauge automated end cutting needle for biopsy. Aspiration was carried out using either an 18-gauge Franssen needle or 21G Chiba needle in a coaxial needle-like manner. These procedures were carried out by three interventional radiologists with 3–17 years operation experience.

Post-procedure, a low-dose CT chest scan was undertaken to identify potential complications, such as pneumothorax or pulmonary hemorrhage, before the patient's departure from the operating room. When a stable and limited pneumothorax is present, it is feasible to carry out an X-ray examination within 24 h following biopsy, thereby allowing for close observation without necessitating immediate intervention. However, if the pneumothorax progressed or the patient's condition worsened, aspiration or chest drain insertion was deemed necessary to avoid further complications.

### Statistical analysis

Baseline characteristics were summarized using descriptive statistics. Continuous variables were presented as median ± standard deviation (SD) or median (interquartile range), while categorical variables were expressed as the number and percentage. To identify significant differences among different NTB, we employed Pearson Chi-square test for categorical variables (or Fisher's exact test as required), one-way ANOVA for normally distributed data, or Kruskal–Wallis H tests for continuous variables.

To investigate the association between NTB and postoperative immediate pneumothorax, we constructed univariate and multivariate binary logistic regression models, in accordance with the Strengthening the Reporting of Observational Studies in Epidemiology (STROBE) statement guidelines^13^.

We constructed three distinct logistic regression models. The non-adjusted Model did not adjust for any potential confounders. The minimally adjusted Model controlled for age, sex, height, weight, chronic obstructive pulmonary disease (COPD), smoking status, alcohol consumption, systolic and diastolic blood pressure. The fully adjusted model further adjusted for the operator's experience, patient's puncture position, lesion located in lung, lesion located in lobes, lesion diameter, transpulmonary needle path length, entire needle path length, needle-pleura angle, puncture through interlobar fissure, number of pleural punctures, number of needle redirections, operation duration, puncture needle gauge, pathological results, and needle path CT attenuation values. The coefficient of association (odds ratio, OR) and 95% confidence intervals (95% CI) were reported.

We evaluated the correlation between NTB and immediate postoperative pneumothorax on a continuous scale employing restricted cubic spline curves based on a fully adjusted logistic regression model. To strike a balance between optimal fit and overfitting, we constructed a model with four knots at the 5th, 35th, 65th, and 95th percentiles of NTB (with the 5th percentile serving as the reference), consistent with Harrell recommendation^15^. The Wald test was used to test the non-linearity^16^. If a nonlinear relationship was identified, the inflection point was subsequently calculated using a recursive algorithm and bootstrapping^17^. Subsequently, a two-piece binary logistic regression model was used to generate a segmented linear model both sides of the inflection point. Finally, we presented the OR along with the 95% CI.

To validate the correlation between NTB and postoperative immediate pneumothorax, the continuous variable of NTB was transformed into categorical variable based on quartiles. We calculated the P for trend to validate the results obtained when treating NTB as a continuous variable and to investigate the possibility of non-linearity. We further examined the potential impact of unknown confounders on the relationship between NTB and the rate of postoperative immediate pneumothorax by calculating E-values^18^. Additionally, to create a more consistent cohort and minimize the influence of potential confounding variables, we excluded from our analysis those cases without NTB.

The missing values for height was 10 (2.20%), weight 7 (1.50%), SBP 5 (1.10%), DBP 5 (1.10%), alcohol consumption 4 (0.90%), and smoking status 3 (0.70%), respectively. Given that the missing data for all variables were less than 8%, these cases were excluded from the regression models.

The statistical analyses were conducted using R software version 4.2.3 (R Foundation for Statistical Computing, Vienna, Austria; https://www.r-project.org/), and two-sided p value < 0.05 was considered statistically significant.

## Results

### Baseline characteristics of the participants

Among the 674 subjects who underwent CT-PTLB, 221 were excluded, leaving 453 participants (47.90% male) who met the inclusion criteria for this study (as shown in Fig. 1).

Table 1 presents the baseline characteristics of the participants, categorized by quartiles of NTB: Q1: (0–1) mm, Q2 (2–3) mm, Q3 (5–8) mm, and Q4 (14–26) mm. The median NTB among all participants was 4 mm (ranging from 0 to 78 mm). The postoperative immediate pneumothorax rate of included patients was 41.06% (186/453). When comparing the lowest and highest NTB quartiles, it was noted that those in the top quartile were non-smokers, taller, older, more likely to be male, and with larger needle diameter. Additionally, larger lesion diameters, longer and deeper needle paths (total or transpulmonary), larger needle-pleura angles, and higher frequency of needle redirection. These participants also experienced longer operation times, higher needle path CT attenuation, and were more likely to show malignant pathological results. Notably, the incidence of immediate pneumothorax decreased with an increase in NTB.

**Table 1:** The baseline characteristics by quartiles of needle track bleeding.

NTB (Q1, Q3) (mm)	Q1 (0, 1)	Q2 (2, 3)	Q3 (5, 8)	Q4 (14, 26)	*P*-value
	N = 114	N = 113	N = 113	N = 113	
COPD, N (%)					0.677
No	111 (97.4%)	108 (95.6%)	110 (97.3%)	107 (94.7%)	
Yes	3 (2.63%)	5 (4.42%)	3 (2.65%)	6 (5.31%)	
Smoking status, N (%)					0.020
Never	82 (73.2%)	84 (74.3%)	68 (60.2%)	61 (54.5%)	
Former	14 (12.5%)	16 (14.2%)	20 (17.7%)	24 (21.4%)	
Current	16 (14.3%)	13 (11.5%)	25 (22.1%)	27 (24.1%)	
Alcohol consumption, N (%)					0.144
Never	84 (74.3%)	83 (73.5%)	72 (64.9%)	73 (65.2%)	
Former	12 (10.6%)	20 (17.7%)	24 (21.6%)	18 (16.1%)	
Current	17 (15.0%)	10 (8.85%)	15 (13.5%)	21 (18.8%)	
SBP, median (Q1, Q3) (mmHg)	125 (112, 139)	127 (116, 140)	130 (116, 141)	132 (120, 146)	0.056
DBP, Mean (SD) (mmHg)	79.0 (9.53)	80.8 (10.5)	81.0 (10.6)	81.0 (10.2)	0.404
Height, median (Q1, Q3) (cm)	160 (155, 165)	160 (155, 168)	162 (160, 168)	163 (158, 169)	0.002
Weight, median (Q1, Q3) (Kg)	60.0 (54.0, 66.0)	60.0 (54.0, 68.0)	60.0 (52.0, 69.0)	61.0 (55.0, 70.0)	0.551
Age, median (Q1, Q3) (Years)	60.0 (50.2, 68.0)	57.0 (51.0, 69.0)	61.0 (52.0, 70.0)	65.0 (56.0, 71.0)	0.029
Sex, N (%)					< 0.001
Male	40 (35.1%)	45 (39.8%)	65 (57.5%)	67 (59.3%)	
Female	74 (64.9%)	68 (60.2%)	48 (42.5%)	46 (40.7%)	
Operators experience, N (%) (Years)					0.102
> 10	64 (56.1%)	67 (59.3%)	80 (70.8%)	77 (68.1%)	
5–10	44 (38.6%)	43 (38.1%)	32 (28.3%)	31 (27.4%)	
≤ 5	6 (5.26%)	3 (2.65%)	1 (0.88%)	5 (4.42%)	
Needle gauge, N (%) (Gauge)					< 0.001
> 17 G	96 (84.2%)	93 (82.3%)	44 (38.9%)	20 (17.7%)	
≤ 17G	18 (15.8%)	20 (17.7%)	69 (61.1%)	93 (82.3%)	
Patient puncture position, N (%)					< 0.001
Supine	28 (24.6%)	47 (41.6%)	47 (41.6%)	63 (55.8%)	
Prone	46 (40.4%)	43 (38.1%)	59 (52.2%)	46 (40.7%)	
Left lateral position	27 (23.7%)	15 (13.3%)	2 (1.77%)	2 (1.77%)	
Right lateral position	13 (11.4%)	8 (7.08%)	5 (4.42%)	2 (1.77%)	
Lesion located in lung, N (%)					0.895
Left	43 (37.7%)	48 (42.5%)	46 (40.7%)	47 (41.6%)	
Right	71 (62.3%)	65 (57.5%)	67 (59.3%)	66 (58.4%)	
Lesion located in lobes, N (%)					0.502
Upper	67 (58.8%)	68 (60.2%)	71 (62.8%)	80 (70.8%)	
Lingual / Middle	3 (2.63%)	4 (3.54%)	3 (2.65%)	4 (3.54%)	
Lower	44 (38.6%)	41 (36.3%)	39 (34.5%)	29 (25.7%)	
Immediate pneumothorax, N (%)					< 0.001
No	41 (36.0%)	57 (50.4%)	80 (70.8%)	89 (78.8%)	
Yes	73 (64.0%)	56 (49.6%)	33 (29.2%)	24 (21.2%)	
Lesion diameter, median (Q1, Q3) (cm)	1.00 (0.90, 1.20)	1.10 (0.90, 1.50)	1.90 (1.00, 2.80)	2.20 (1.50, 3.20)	< 0.001
Entire needle path length median (Q1, Q3) (cm)	5.60 (4.73, 6.70)	6.10 (5.10, 7.00)	6.40 (5.10, 7.70)	6.80 (5.90, 8.20)	< 0.001
Transpulmonary needle path length median (Q1, Q3) (cm)	1.50 (1.00, 2.00)	1.80 (1.20, 2.40)	1.80 (1.30, 2.50)	2.70 (1.90, 3.80)	< 0.001
Needle-pleura angle, median (Q1, Q3) (Degree)	73.0 (57.0, 82.0)	73.0 (61.0, 84.0)	65.0 (50.0, 80.0)	67.0 (54.0, 80.0)	0.041
Puncturing though interlobar fissure, N (%)					0.940
No	113 (99.1%)	112 (99.1%)	112 (99.1%)	111 (98.2%)	
Yes	1 (0.88%)	1 (0.88%)	1 (0.88%)	2 (1.77%)	
Number of pleural punctures, N (%)					0.062
1	110 (96.5%)	110 (97.3%)	113 (100%)	105 (92.9%)	
2	4 (3.51%)	3 (2.65%)	0 (0.00%)	6 (5.31%)	
3	0 (0.00%)	0 (0.00%)	0 (0.00%)	1 (0.88%)	
4	0 (0.00%)	0 (0.00%)	0 (0.00%)	1 (0.88%)	
Number of needle redirection, median (Q1, Q3)	3.00 (2.00, 4.75)	3.00 (3.00, 4.00)	4.00 (3.00, 5.00)	6.00 (4.00, 8.00)	< 0.001
Operation duration, median (Q1, Q3) (Minutes)	7.00 (5.00, 11.0)	7.00 (5.00, 11.0)	11.0 (7.00, 15.0)	18.0 (12.0, 24.0)	< 0.001
Intraoperative pneumothorax, N (%)					0.955
No	104 (91.2%)	103 (91.2%)	101 (89.4%)	103 (91.2%)	
Yes	10 (8.77%)	10 (8.85%)	12 (10.6%)	10 (8.85%)	
Delayed pneumothorax N (%)					0.271
No	113 (99.1%)	112 (99.1%)	110 (97.3%)	108 (95.6%)	
Yes	1 (0.88%)	1 (0.88%)	3 (2.65%)	5 (4.42%)	
Pathological results, N (%)					0.022
Benign	45 (39.5%)	31 (27.4%)	27 (23.9%)	26 (23.0%)	
Malignant	69 (60.5%)	82 (72.6%)	86 (76.1%)	87 (77.0%)	
Along needle path CT attenuation median (Q1, Q3) (Hu)	− 851.00 (− 869.00, − 822.50)	− 835.00 (− 867.00, − 808.00)	− 817.00 (− 848.00, − 796.00)	− 814.00 (− 848.00, − 783.00)	< 0.001
Hospital stays, median (Q1, Q3) (Days)	11.0 (8.00–14.0)	12.0 (9.00–17.0)	12.0 (9.00–17.0)	11.0 (7.00–17.0)	0.112

Q1, Q2, Q3 and Q4 represent four quartiles of NTB.

*NTB* needle track bleeding, *COPD* chronic obstructive pulmonary disease, *SBP* systolic blood pressure, *DBP* diastolic blood pressure, *N* number, *Hu* Hounsfield unite.

### The results of univariate analysis using binary logistic regression model

To explore potential confounding factors and the relationship between the NTB and immediate postoperative pneumothorax, a univariate analysis was conducted. The findings suggested that needle path CT attenuation, patient weight, smaller needle diameter, lesion diameter, entire needle path length, and NTB negatively correlated with the occurrence of immediate pneumothorax. Conversely, age, lateral position during procedure, and number of pleural punctures were positively associated with postoperative immediate pneumothorax (Supplementary Table S1).

### The results of multivariate analysis using the binary logistic regression model

We used three model to explore the link between NTB and postoperative immediate pneumothorax (Table 2). When considering NTB as a continuous variable, linear regression was observed. In the unadjusted model, we found that an increase of 1 mm width of NTB was related to 7% decrease in postoperative immediate pneumothorax (OR = 0.93, 95% CI 0.90–0.96), which was statistically significant. A similar pattern was observed in the minimally adjusted model, with an 8% decrease in the pneumothorax incidence for 1 mm increase in NTB width (OR = 0.92, 95% CI 0.90–0.95). The fully adjusted model, which accounted for all confounders, reinforced these findings. 1 mm increase in NTB width was associated with a 9% decrease in postoperative pneumothorax risk (OR = 0.91, 95% CI 0.88–0.95).

**Table 2:** The relationship between needle track bleeding and postoperative immediate pneumothorax in the three binary logistic regression models.

Exposure	Non-adjusted Model OR, (95% CI), *P*-value	Minimally adjusted Model OR, (95% CI), *P*-value	Full adjusted Model OR, (95% CI), *P*-value
NTB (mm)	0.93 (0.90–0.96) < 0.001	0.92 (0.90–0.95) < 0.001	0.91 (0.88–0.95) < 0.001
NTB quartiles			
Q1(0–1.0)	Ref	Ref	Ref
Q2(1.0–4.0)	0.53 (0.31–0.91) 0.021	0.52 (0.29–0.91) 0.021	0.57 (0.31–1.05) 0.070
Q3(4.0–10.0)	0.23 (0.13–0.40) < 0.001	0.19 (0.10–0.35) < 0.001	0.19 (0.09–0.40) < 0.001
Q4(10.0–78.0)	0.15 (0.08–0.27) < 0.001	0.11 (0.06–0.22) < 0.001	0.08 (0.03–0.19) < 0.001
*P* for trend	< 0.001	< 0.001	< 0.001

Q1, Q2, Q3 and Q4 represent four quartiles of NTB.

*NTB* needle track bleeding, *OR* odds ratio, *CI* confidence intervals, *Ref* reference, *COPD* chronic obstructive pulmonary disease.

Non-adjusted Model: not adjusted for any covariates.

Minimally adjusted Model: adjusted for COPD, smoking status, alcohol consumption, systolic and diastolic blood pressure, height, weight, age, sex.

Fully adjusted Model: further adjusted for operator's experience, patient's puncture position, lesion located in lung, lesion located in lobes, lesion diameter, puncture needle gauge, transpulmonary needle path length, number of pleural punctures, entire needle path length, number of needle redirections, operation duration, pathological results, and needle path CT attenuation values, needle-pleura angle, puncture through interlobar fissure.

### Sensitivity analysis

We executed sensitivity analysis to evaluate the robustness of our findings. Initially, when NTB were used as quartiles categorical variable, we found statistically significant differences in the NTB level groups of three models (*p* < 0.001). Compared with NTB Q1 group, the NTB Q4 group could decrease the risk of postoperative immediate pneumothorax by 92% (OR = 0.08, 95% CI 0.03–0.19) in fully adjusted model. Additionally, the P for trend showed *p* < 0.001 in the three models which is consistent with the results when NTB was treated as a continuity variable.

Subsequently, to evaluate the influence of potential unmeasured confounders, we calculated an E-value of 1.43. This exceeded the relative risk of unobserved confounders affecting the association between NTB and postoperative immediate pneumothorax, implying a minimal impact from unknown or unmeasured confounders on this relationship.

Lastly, we ruled out the cases without NTB and then reanalysis the data of 371 participants. The postoperative immediate pneumothorax was 35.58% (132/371). We observed a similar significant association between NTB and postoperative immediate pneumothorax in three binary logistic regression models (Supplementary Table S2 for details).

### Curve fitting and analysis of inflection point

**Figure 3:**
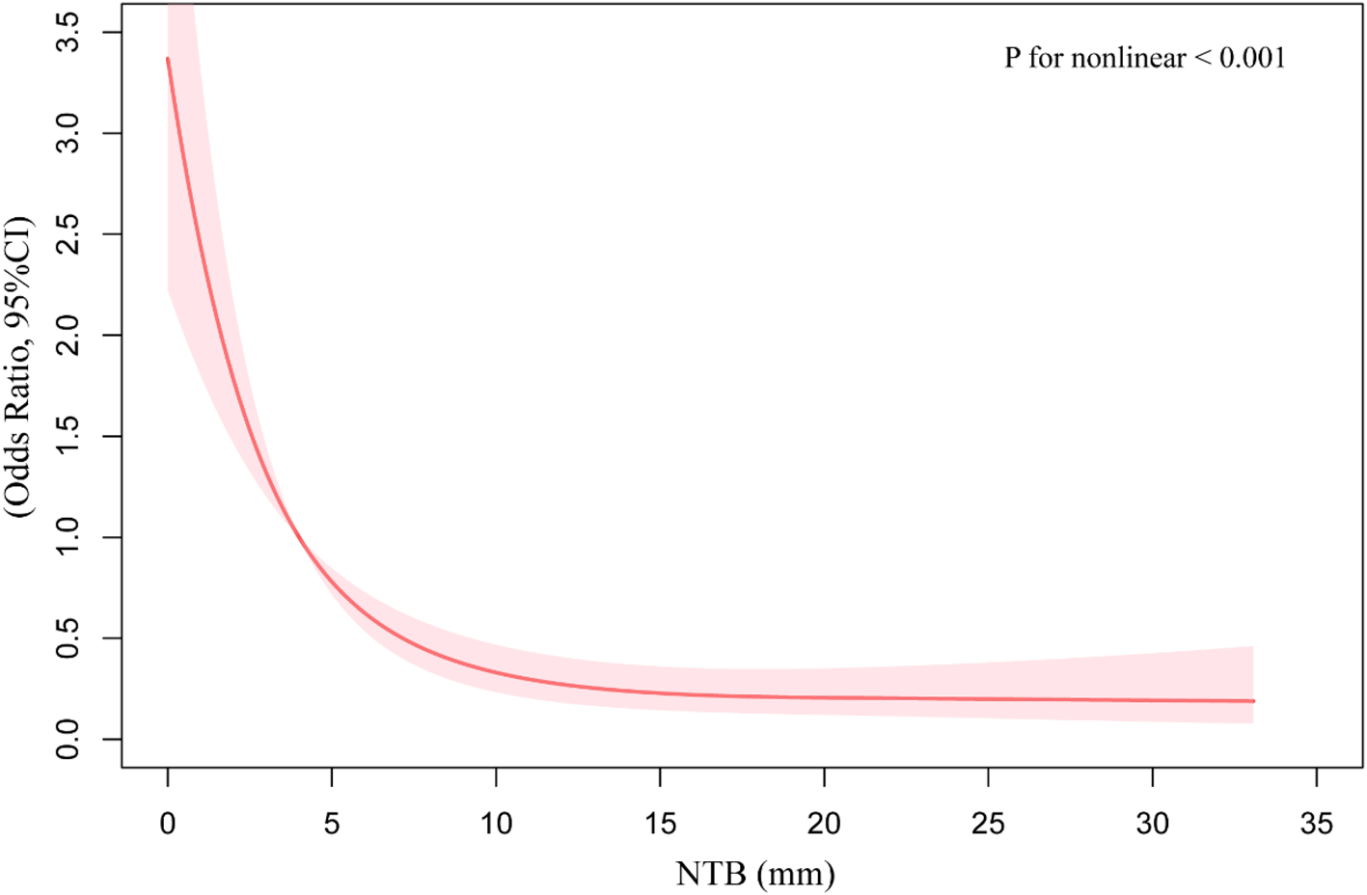
The non-linear association between needle track bleeding and postoperative immediate pneumothorax using a full adjusted restricted cubic spline regression model. Red solid lines indicate OR, and shadow shape indicate 95% CI. *OR* odds ratio, *CI* confidence interval, *NTB* needle tack bleeding.

We found that the relationship between NTB and postoperative immediate pneumothorax was non-linear, and an inflection point was observed through restricted cubic spline curves based on a full adjusted confounders model (p for nonlinear < 0.001, Fig. 3).

In our study, we utilized a piecewise binary logistic regression model to analyze the data, in addition to a standard binary logistic regression model, and the best-fit model was chosen through the log-likelihood ratio test. The P for log-likelihood ratio test was < 0.001 in our analysis (shown in Table 3). So, a piecewise model was used to fit the association between NTB and postoperative immediate pneumothorax. With recursive algorithm, an inflection point (NTB = 8 mm) was identified. When NTB ≤ 8 mm, there was a notable negative association with immediate postoperative pneumothorax (OR = 0.74, 95% CI 0.66–0.81, *p* < 0.001). However, when NTB > 8 mm, no significant relationship with immediate pneumothorax was observed (OR = 0.98, 95% CI 0.95–1.02, *p* = 0.428) (Refer to Table 3).

**Table 3:** The results of the two piecewise linear regression models.

Outcome	OR (95% CI) *P*-value
Fitting by standard linear regression	0.91 (0.88–0.95) < 0.001
Fitting by two-piecewise linear regression	
Inflection point (mm)	8
≤ 8	0.74 (0.66–0.81) < 0.001
> 8	0.98 (0.95–1.02) 0.428
*P* for log-likelihood ratio test	< 0.001

*OR* odds ratio, *CI* confidence intervals, *COPD* chronic obstructive pulmonary disease.

Adjusted for COPD, smoking status, alcohol consumption, systolic and diastolic blood pressure, height, weight, age, sex, operator's experience, patient's puncture position, lesion located in lung, lesion located in lobes, lesion diameter, puncture needle gauge, transpulmonary needle path length, number of pleural punctures, entire needle path length, number of needle redirections, operation duration, pathological results, and needle path CT attenuation values, needle-pleura angle, puncture through interlobar fissure.

## Discussion

Our study shows that NTB is a strong predictor of postoperative immediate pneumothorax in CT-TPLB. Specifically, we report a novel non-linear relationship between NTB distance and postoperative immediate pneumothorax, with a crucial inflection point at 8 mm. For NTB ≤ 8 mm, each 1 mm increase correlates with a 26% decrease in pneumothorax risk (OR = 0.74, 95% CI 0.66–0.81). However, NTB > 8 mm show no significant association with postoperative immediate pneumothorax (P-value > 0.05), suggesting that severity of NTB does not influence the risk beyond this point. Therefore, a NTB of 8 mm is a useful indicator to predict postoperative immediate pneumothorax in adult patients with CT-TPLB. In clinical practice, these findings can be used to identify high-risk patients who may benefit from specialized care.

Patient undergoing CT-TPLB are prone to pneumothorax due to various factors, such as patient-related (age, BMI, emphysematous lungs, bulla), lesion and operation related (position, needle diameter, fissure crossed, multiple pleural punctures, lesion size, transpulmonary length, operator experience)^19,20^.

It has been demonstrated that patients with NTB show a reduced risk of pneumothorax. Prior research indicated that NTB act as a protective factor against pneumothorax^10^. This suggests that identifying NTB may have a predictive value for pneumothorax incidents in patients CT-TPLB. The relationship between NTB and postoperative pneumothorax prevalence has been studied. Recent research found that NTB can contribute to a lower likelihood of pneumothorax^12^. Our study in line with the above studies, but these studies failed to quantitative analysis of the relationship between NTB and pneumothorax.

Building on observations of NTB is against post biopsy pneumothorax occurrence, it appears plausible that creating an artificial along-needle track bleeding could serve as a valuable measure to reduce the incidence of pneumothorax and other complications. This strategy involves the intentional injection of patch materials along the biopsy track during needle withdrawal to simulate the protective effect of NTB. A ground-breaking study by Ronald McCartney^21^ validated this idea in both patient and animal models, demonstrating that pneumothorax could be virtually eliminated as a procedural complication via a lung patch technique. But the injection width of patch materials is few reported.

Massimo De Filippo et al.^10^ along with Vittorio Sabatino et al.^11^ use 6 mm as the cut-off value for classifying NTB, In their study, compared to NTB < 6 mm, the higher NTB group (NTB > 6 mm) reduced pneumothorax, It means that a certain amount of NTB will reduce postoperative pneumothorax. Based on our research result the inflection point of NTB is 8 mm, which is inconsistent with the previous research. In our study, we initially performed linear regression on the adjusted model and considered the comprehensively confounder. Subsequently, we established an association using curve fitting and found a clinical saturation effect and a meaningful inflection point. Further, a sensitivity analysis of trend test in the linear model demonstrated that curve fitting was better than linear fitting to explain the association between NTB and postoperative immediate pneumothorax. So, the cut-off value of NTB in our study derived through strict statistical inference, rather than arbitrarily defined.

Furthermore, this optimal cut-off value is great clinical significance, due to help predict the occurrence of postoperative pneumothorax and providing reference to guide effective biopsy track sealing with various path materials^22–25^, especially for autologous blood seal.

The mechanism of NTB reducing the occurrence of pneumothorax remain unclear, however, several factors might provide a plausible explanation. (1) Self-sealing Effect: along needle track alveolar presence of blood products might serve as a natural self-sealing agent, effectively preventing or reducing air-leak into pleural cavity^21^. (2) Induction of Clotting: hemorrhage along the needle path could initiate clotting, resulting in a patch-like formation that may limit airflow from the airspace to the pleural space^10^. (3) Altered Ventilation: the presence of blood products within the alveolar space could lead to a reduction in ventilation, subsequently decreasing the flow of air from the airspace to the pleural space and reduce the risk of pneumothorax. (4) Promotion of Pleural Adhesion: blood accumulation on the pleural surface might stimulate the adhesion between the visceral and parietal pleura, thus preventing the formation of air pockets that could lead to pneumothorax. (5) Increased Airway Pressure: blood in the alveolar space could increase airway pressure. This increased pressure might counteract the forces causing air to leak from the lungs, thereby reducing the occurrence of pneumothorax. (6) Altering Lung Compliance: Pulmonary hemorrhage may change the lung compliance. Reduced lung compliance means the lungs are less likely to overexpand and cause an air leak, thus reducing the risk of pneumothorax. While these proposed mechanisms offer some insights into the relationship between NTB and reduced pneumothorax incidence, further research are needed for definitive evidence.

Our study has some strengths as follows: (1) To the best of our knowledge, this is the first study to observe and quantify the association between NTB and postoperative immediate pneumothorax using confounder adjusted multiple models. (2) We found a nonlinear relationship between them, and an optimal inflection point was identified. Thus, our study has greater clinical significance, which previous studies have not investigated. (3) We used sensitivity analysis to test the robustness of our findings. (4) Try our best to offer plausible mechanisms for the protective effect of NTB.

Our research has limitations: Firstly, this was a retrospective study, lack of a prospective evaluation and the absence of long-term follow-up in patients who developed NTB, further validation in prospective studies should be considered. Secondly, due to the observational nature, residual or unmeasured confounding factors might interfere with the results. However, we computed the E-value to quantify the potential influence of unmeasured confounders and conclude that they were unlikely to influence the findings. Thirdly, we couldn't quantify pneumothorax volume. We categorized patients into pneumothorax and no pneumothorax groups following the CT scanner, and thus the delayed pneumothorax was assessed mostly by X-ray. This might not achieve accurate grouping or underestimate delayed pneumothorax rate, and we failed to analyze the association between NTB and delayed pneumothorax because only 10 patients were detected. Fourthly, being a single-center study from China, our findings may not be generalizable to other settings. Lastly, we didn't measure NTP volume or consider other types of pulmonary hemorrhage (such as lesion perilesional bleeding, hemoptysis), focusing on NTB, the relationship between other types of pulmonary hemorrhage and pneumothorax needs further study. Despite these potential limitations, our study was the first to reveal NTB associated with immediate pneumothorax risk in a nonlinear relationship with different regression models, and the conclusions postulated remain credible.

## Conclusion

Our study reveals that NTB were nonlinearly associated with postoperative immediate pneumothorax, with an optimal inflection point, and the NTB was a protective factor of postoperative immediate pneumothorax. A significant negative association with postoperative immediate pneumothorax was discovered when NTB ≤ 8 mm, these finding are expected to provide suitable guidance for prognosis prediction and guiding sealant application.

### Supplementary Information

Supplementary Tables.

## Data Availability

The data that support the findings of this study are available from the corresponding author upon reasonable request. The data are not publicly available due to privacy or ethical restrictions.

## Abbreviations

COPD: Chronic obstructive pulmonary disease
SBP: Systolic blood pressure
DBP: Diastolic blood pressure
BMI: Body mass index
CT-PTLB: Computed tomography-guided percutaneous transthoracic lung biopsy
OR: Odds ratio
CI: Confidence intervals
PH: Pulmonary hemorrhage
NTB: Needle track bleeding
