# Supplementary material for: Association between needle track bleeding and postoperative immediate pneumothorax in CT-guided percutaneous transthoracic lung biopsies: a cross-sectional study
Source: Sci Rep. 2023 Nov 1;13:18811. doi: 10.1038/s41598-023-44560-2 (PMC10620196; doi:10.1038/s41598-023-44560-2)
Supplement: Supplementary file 1 — Supplementary Tables. [file 41598_2023_44560_MOESM1_ESM.docx]

Supplementary Materials

**Table S1** The results of the univariate analysis

| Variable | Statistics | OR (95%CI) *P*-value |
| --- | --- | --- |
| Needle path CT attenuation values, mean ± SD (Hu) | -827 ± 49.42 | 0.99 (0.98 - 0.99) < 0.001 |
| COPD, N (%) |  |  |
| No | 16 (3.64 %) | Ref |
| Yes | 423 (96.36 %) | 1.12 (0.41 - 3.08) 0.820 |
| Smoking status, N (%) |  |  |
| Never | 80 (18.22 %) | Ref |
| Former | 287 (65.38 %) | 0.87 (0.51 - 1.48) 0.615 |
| Current | 72 (16.4 %) | 0.87 (0.52 - 1.44) 0.584 |
| Alcohol consumption, N (%) |  |  |
| Never | 74 (16.86 %) | Ref |
| Former | 303 (69.02 %) | 0.86 (0.51 - 1.44) 0.557 |
| Current | 62 (14.12 %) | 1.01 (0.58 - 1.77) 0.959 |
| SBP, mean ± SD (mmHg) | 128.95 ± 18.25 | 1.00 (0.99 - 1.01) 0.490 |
| DBP, mean ± SD (mmHg) | 80.44 ± 10.18 | 1.00 (0.98 - 1.01) 0.645 |
| Height, mean ± SD (cm) | 161.9 ± 7.63 | 0.98 (0.95 – 1.00) 0.086 |
| Weight, mean ± SD (Kg) | 61.69 ± 10.68 | 0.98 (0.96 – 1.00) 0.028 |
| Age, mean ± SD (Years) | 60.31 ± 12.03 | 1.02 (1.00 - 1.04) 0.020 |
| Sex, N (%) |  |  |
| Male | 211 (48.06 %) | Ref |
| Female | 228 (51.94 %) | 1.19 (0.81 - 1.74) 0.381 |
| Experience, N (%) (Years) |  |  |
| ≤ 5 | 278 (63.33 %) | Ref |
| 5-10 | 147 (33.49 %) | 1.23 (0.82 - 1.84) 0.318 |
| > 10 | 14 (3.19 %) | 1.16 (0.39 - 3.44) 0.785 |
| Puncture needle gauge, N (%) (Gauge) |  |  |
| ≤ 17 G | 192 (43.74 %) | Ref |
| > 17G | 247 (56.26 %) | 0.61 (0.41 - 0.9) 0.013 |
| Patient puncture position, N (%) |  |  |
| Supine or prone | 369 (84.05 %) | Ref |
| Other | 70 (15.95 %) | 4.24 (2.44 - 7.38) < 0.001 |
| Lung, N (%) |  |  |
| Left | 177 (40.32 %) | Ref |
| Right | 262 (59.68 %) | 0.98 (0.67 - 1.45) 0.933 |
| Lobes, N (%) |  |  |
| Upper | 14 (3.19 %) | Ref |
| Lingual / middle | 274 (62.41 %) | 2.02 (0.68 - 5.98) 0.205 |
| Lower | 151 (34.4 %) | 1.08 (0.72 - 1.62) 0.696 |
| Lesion diameter, mean ± SD | 1.82 ± 1.23 | 0.85 (0.72 – 1.00) 0.047 |
| Transpulmonary needle path length, mean ± SD (cm) | 2.18 ± 1.29 | 0.93 (0.80 - 1.08) 0.370 |
| Entire needle path length, mean ± SD (cm) | 6.42 ± 1.73 | 0.89 (0.79 - 0.99) 0.038 |
| Needle-pleura angle, mean ± SD (Degree) | 66.65 ± 17.63 | 1.00 (0.99 - 1.01) 0.870 |
| Puncturing though interlobar fissure |  |  |
| No | 5 (1.14 %) | Ref |
| Yes | 434 (98.86 %) | 5.86 (0.65 - 52.9) 0.115 |
| Number of punctures pleural, mean ± SD | 1.04 ± 0.24 | 4.89 (1.40 - 17.06) 0.013 |
| Number of needle redirections, mean ± SD | 4.69 ± 2.69 | 0.96 (0.89 - 1.03) 0.218 |
| Operation duration, mean ± SD (Minutes) | 11.86 ± 7.91 | 0.99 (0.96 - 1.01) 0.270 |
| NTB, mean ± SD (mm) | 7.74 ± 10.24 | 0.93 (0.90 - 0.96) < 0.001 |
| Pathological results, N (%) |  |  |
| Benign | 125 (28.47 %) | Ref |
| Malignant | 314 (71.53 %) | 0.84 (0.55 - 1.28) 0.421 |

Abbreviations: Hu: Hounsfield unite; COPD: Chronic Obstructive Pulmonary Disease; SBP: Systolic Blood Pressure; DBP: Diastolic Blood Pressure; NTB: Needle Track Bleeding; OR: Odds Ratio; CI: Confidence Interval; SD: Standard Deviation; N: Number; refer: Reference.

**Table S2** The multivariate analysis conducted via three logistic regression models ruled out the cases without needle track bleeding.

| Exposure | N | Non-adjusted Model  OR, (95% CI), *P*-value | Minimally adjusted Model  OR, (95% CI), *P*-value | Full adjusted Model  OR, (95% CI), *P*-value |
| --- | --- | --- | --- | --- |
| NTB (mm) | 371 | 0.95 (0.92-0.98) < 0.001 | 0.94 (0.91-0.97) < 0.001 | 0.93 (0.89-0.97) < 0.001 |
| NTB quintiles (mm) |  |  |  |  |
| Q1(0-1.0) | 93 | Ref | Ref | Ref |
| Q2(1.0-4.0) | 93 | 0.60 (0.33-1.06) 0.079 | 0.61 (0.33-1.11) 0.106 | 0.62 (0.31-1.23) 0.174 |
| Q3(4.0-10.0) | 93 | 0.17 (0.09-0.33) < 0.001 | 0.14 (0.07-0.29) < 0.001 | 0.15 (0.06-0.34) < 0.001 |
| Q4(10.0-78.0) | 92 | 0.22 (0.12-0.42) < 0.001 | 0.18 (0.09-0.36) < 0.001 | 0.13 (0.05-0.32) < 0.001 |
| *P* for trend |  | < 0.001 | < 0.001 | < 0.001 |

Abbreviations: NTB: Needle Track Bleeding; OR: Odds Ratio; CI: Confidence Intervals; Ref: Reference; COPD: Chronic Obstructive Pulmonary Disease.

Non-adjusted Model: not adjusted for any covariates.

Minimally adjusted Model: adjusted for COPD, smoking status, alcohol consumption, systolic and diastolic blood pressure, height, weight, age, sex.

Fully adjusted Model: further adjusted for operator's experience, patient's puncture position, lesion located in lung, lesion located in lobes, lesion diameter, puncture needle gauge, transpulmonary needle path length, number of pleural punctures, entire needle path length, number of needle redirections, operation duration, pathological results, and needle path CT attenuation values, needle-pleura angle, puncture through interlobar fissure.
